# Associated factors to pregnancy in intrauterine insemination

**DOI:** 10.5935/1518-0557.20190060

**Published:** 2020

**Authors:** Luis Vargas-Tominaga, Fiorella Alarcón, Andrea Vargas, Gaby Bernal, Andrea Medina, Zarela Polo

**Affiliations:** 1 Centro de Fertilidad y Ginecología del Sur, Cusco, Peru

**Keywords:** intrauterine insemination, IUI, infertility

## Abstract

**Objective:**

To define the factors associated with clinical pregnancy after intrauterine insemination.

**Methods:**

Retrospective study involving 633 infertility couples, which made up to 1053 cycles of intrauterine insemination. We analyzed the clinical pregnancy rate associated with different factors through the Chi-square test or Fisher's exact test.

**Results:**

The clinical pregnancy rate was 8.2% per insemination cycle and 13.6% per treated couple. The factors with greater association to clinical pregnancy were to have more than two follicles, to perform the procedure without difficulty, to have 3 years or less of infertility, cervical factor as indication, use of gonadotropins and age less than 38 years.

**Conclusion:**

Intrauterine insemination requires to be accompanied by proper selection and couples' preparation.

## INTRODUCTION

Intrauterine insemination (IUI) and ovarian stimulation are part of the initial management of couples with infertility, known as low-complexity assisted reproductive technologies (ART). IUI requires basic supplies and simple training, constituting a low-cost technique, accessible for the majority of couples. However, its effectiveness has not been fully researched. Both, patients and the public in our country, believe IUI is very effective. Some researchers consider IUI as a trivial procedure for certain indications and recommend patients to go directly to high complexity ART (^NICE, 2013^). Others, point to it as a procedure with potential complications, specifically associated with multiple pregnancies (^ESHRE Capri Workshop Group, 2009^).

In the Centro de Fertilidad y Ginecología del Sur (CFGS), in the city of Cusco, in the Andes of Peru, IUI has been carried out since 1999. It is important to know which group of patients and conditions are those that enable a greater probability of pregnancy with IUI. The aim of this study was to investigate the parameters, which gave us the best outcomes in more than one thousand IUI cycles.

## MATERIALS AND METHODS

It is a retrospective study, reviewing charts and records of procedures from patients in whom IUI was conducted from January 1999 until April 2018. We include patients with evidence of at least one permeable tube documented through hysterosalpingography or laparoscopy. The semen samples were processed through swim-up or by density gradients.

Either IUI was done around 36 hours of the hCG (human chorionic gonadotropin) injection, prior or subsequent to ovulation, documenting this through vaginal ultrasound, and in some cases, IUI was carried out in both moments, which is two IUI in a single cycle. The IUI was done with different types of catheters, from diverse trademarks, soft or semi-rigid tubes. Prior to IUI, we require informed consent from the patient and her partner.

The clinical pregnancy rate (CPR) is defined as the number of patients with active intrauterine pregnancy in relation to the number of patients in whom IUI was performed. The statistical significance of the differences found were analyzed through the Chi-square test or the Fisher's exact test.

## RESULTS

1053 IUI cycles were performed in 633 patients, achieving positive pregnancy test results in 11.0% of the cycles: 57 live births, 19 patients with ongoing pregnancy at the time of the last evaluation, 22 coursed with abortion, 5 had ectopic pregnancies (one case was a heterotopic pregnancy), 10 with biochemical pregnancy and in 3 we lost contact with the patient after the pregnancy test result. The CPR was 8.2% per IUI cycle and 13.6% per treated couple.

IUI was performed in 33.5% for unexplained infertility, 24.5% for cervical factor, 31.7% for male factor and 10.3% for endometriosis (Table 1). 49.0% were women ≤ 34 years of age, 35.5% between 35 to 39 years and 15.5% ≥40 years of age (Table 1). IUI cycles in women younger than 38 years had a CPR of 9.4%, significantly more than the 5.4% in women with 38 years of age and older (Table 2). We evaluated several factors related to the diagnosis of infertility and the IUI procedure, and its possible association with pregnancy (Table 2), with its corresponding CPR and Odds ratio (OR), and confidence intervals (CI) for those factors with statistical significance (*p*-value).

**Table 1:** Age and indication of IUI

	Frequency	CPR/cycle	CPR/patient
AGE:			
≤ 34 years	49.0%	9.7%	15.5%
35- 39 years	35.5%	8.9%	15.1%
≥ 40years	15.5%	2.3%*	4.1%*
INDICATION			
Unexplained infertility	33.5%	4.5%*	7.6%*
Cervical factor	24.5%	13.5%*	20.7%*
Male factor	31.7%	8.4%	15.4%
Endometriosis	10.3%	7.9%	14.0%

* *p*˂0.05.

**Table 2:** Associated factors

Factor	CPR/cycle	*p*	OR	superior CI	inferior CI
Age <38 years	9.4 *vs.* 5.4%	0.030	1.819	1.052	3.147
Cervical factor	13.5 *vs.* 6.6%	0.001	2.194	1.380	3.488
Infertility ≤3 years	10.2 *vs.* 4.8%	0.003	2.240	1.295	3.875
Infertility I/II	6.8 *vs.* 9.2%	0.175			
IUI cycles ≤2	8.5 *vs.* 5.8%	0.264			
Gonadotropins	9.7 *vs.* 5.5%	0.019	1.846	1.100	3.097
Follicles 1 vs ≥2	4.4 *vs.* 11.6%	<0.00	2.873	1.697	4.864
Pre-cap <5 millions	3.3 *vs.* 8.8%	0.134			
Post-cap <5 millions	5.2 *vs.* 8.6%	0.134			
Post-cap <30 millions	7.4 *vs.* 9.7%	0.194			
Post-cap <50 millions	7.8 *vs.* 10.4%	0.222			
Swim-up/gradients	9.4 *vs.* 8.2%	0.582			
Double/simple IUI	16.7 *vs.* 7.9%	0.088			
Pre/post ovulation	8.2 *vs.* 5.4%	0.146			
Easy/hard IUI	9.7 *vs.* 4.4%	0.014	2.341	1.168	4.693
Endometrium<9 mm	6.5 *vs.* 9.8%	0.058			

A better CPR was found for those patients with a history of infertility for 3 years or less, compared to those with a longer history (10.2% *vs.* 4.8%, *p*=0.003, OR 2.240). A history of primary infertility is associated with a CPR of 6.8%, while secondary infertility to a CPR of 9.2%, without any statistically significant difference. IUI yielded pregnancy in 8.5% of patients on a first or second attempt, while 5.8% in the third or later (Table 3).

**Table 3:** Number of IUI cycles

Number of IUI cycles	CPR
1	8.5%
2	8.6%
3	6.0%
≥4	5.1%

IUI in association with gonadotropins (HMG or FSHr) achieves a significantly higher CPR in comparison to the IUI associated with clomiphene citrate, letrozole, tamoxifen or natural cycle (9.7% *versus* 5.5%, *p*=0.019, OR 1846), and having 2 follicles or more yields a CPR of 11.6%, significantly more than the 4.4% in cases of only having 1 follicle (*p*<0.000, OR 2.873).

Sperm with progressive motility (SPM) concentration both prior and after capacitation showed no significant influences on CPR of IUI. In the sample prior to capacitation, the CPR was 3.3% with concentration less than 5 million of SPM per ml, while it grew to 8.8% when the concentration was greater than 5 million. In the sample after capacitation, the CPR was 5.2% *versus* 8.6% when concentration was less or greater than 5 million per ml of SPM, respectively; 7.4% *versus* 9.7% when concentration was less or more than 30 million; and 7.8% *versus* 10.4% when the concentration was less than or greater than 50 million.

In all our IUI procedures, the semen sample was capacitated through swim-up in 10.3% of cases, and in 89.7% through density gradients, noting that the CPR was similar for both methods (9.4% and 8.2%, respectively). Performing two inseminations in one cycle yields a CPR of 16.7%, compared to 7.9% for single inseminations; however, the differences were not statistically significant (*p*=0.088). There were no statistically significant differences in CPR by IUI performed before or after ovulation (8.2% *versus* 5.4%, *p*=0.146). CPR decreased when the IUI was conducted with difficulty, which is 4.4%, compared to the 9.7% when the procedure is easy.

## DISCUSSION

IUI is part of the initial infertility management in couples, and many researchers demonstrate acceptable pregnancy rates. Farquhar *et al*. (2017) reported CPR of 31% for IUI compared to 9% of spontaneous evolution, without treatment, and Bensdorp *et al*. (2015) found that IUI with ovarian stimulation, IVF with single embryo transfer and IVF in modified natural cycle have similar success rates (live born rate of 47%, 52% and 43%, respectively).

However, regardless of the excellent CPR reported by these studies, data that grouped international reports, such as the Latin American Network of Assisted Reproduction (REDLARA), found a CPR of 14.91% for the year 2013 (Zegers-Hochschild *et al.,* 2016), and the world report of the International Committee for Monitoring Assisted Reproductive Technologies (ICMART) found a CPR of 12.1% for the year 2010 (Dyer *et al*., 2016). Our study has a CPR of 8.2% per cycle.

In relation to age, we found 38 years of age to be determinant, with CPR of 9.4% in women <38 years compared to 5.6% in women ≥38 years. Some studies consider 40 years as crucial for the CPR (Ashrafi *et al*., 2013; Nuojua-Huttunen *et al*., 1999); and others consider 35 years to be determinant, such as Sicchieri *et al*. (2018), who describes a CPR of 12.7% in women <35 years and 7.6% in the total group. REDLARA reports a CPR of 18.4% for women < 35 years, 13.4% between 35 to 39 years, 7.1% between 40 to 42 years, and 3.5% in women <42 years (^Zegers-Hochschild *et al*., 2016^).

Upon assessing SPM concentration, we did not find significant differences in CPR, both when considering the previous sample and for further capacitation. Irani *et al*. (2018) considers transcendent total number of SPM prior to capacitation, and found patients with SPM concentration <20 million to have CPR of 17.8% compared to 4.6% when the concentration is lower. Kuriya *et al*. (2018) considers the total number of SPM prior to capacitation as the only important semen parameter, having higher CPR when it is <39 million. Punjabi *et al*. (2018) found the number of inseminated SPM as the main factor, reporting that among over 2 million subjects, the CPR was 13.8% in comparison to 4.4% when the number is lower. The capacitation method did not change the CPR in our study. A meta-analysis from Boomsma *et al*. (2007) did not find differences.

When two inseminations are carried out in one cycle, better CPR is achieved; however, the differences were not statistically significant. Cantineau *et al*. (2003) and Ragni *et al*. (1999) reported a better CPR with double IUI. Others, such as Zahiri Sorouri *et al*. (2016), Tonguc *et al*. (2010) and Alborzi *et al*. (2003) did not find differences between simple and double IUI. Gonadotropins in association with IUI yielded a better CPR, a finding that is in agreement with what has been reported by other investigators, such as Banker *et al*. (2018), who reported a CPR of 14.6% for IUI in association with gonadotropins (with or without clomiphene citrate), while CPR is 7.8% when associated with clomiphene citrate alone; or Cabry-Goubet *et al*. (2017), who reported a CPR between 12.7% to 14.2% when associating IUI to gonadotropins.

In our study, having two follicles or more was the most important factor associated to CPR (11.6% with ≥2 follicles and 4.4% with <2 follicles, OR 2.283), a finding also reported in other studies. Irani *et al*. (2018) reported 20 million SPM prior to capacitation as determinant in the CPR; however, if the response to clomiphene citrate is ≥2 follicles and with endometrial thickness greater than 7 mm, the necessary number of SPM for a satisfactory CPR reduces to 10 million. Van Rumste *et al*. (2008) performed a meta-analysis and reported the IUI associated to monofolicular and multifolicular cycles, finding at first a CPR of 8.4%; while when having 2, 3 or 4 follicles raised the CPR to 5, 8, and 8%, respectively.

Our study did not find significant CPR differences when IUI was done before or after ovulation. Wang *et al*. (2006) did not find differences in the CPR doing IUI 24 or 36 hours of hCG use; Huang *et al*. (2000) also did not find differences doing IUI 26-28 hours or 36-38 hours of hCG injection; and a meta-analysis by Cantineau *et al*. (2014) had the same outcome. Lee *et al*. (2018) reported lower CPR when performing IUI before 36 hours of ovulation (5%); compared to 36-37 hours, 37-38 hours and more than 38 hours (21.8% *vs.* 24.8% *vs.* 20.0%), emphasizing that the first group had a lower percentage of normal sperm morphology, and Rahman *et al*. (2011) reported less CPR doing IUI 24 hours after administration of hCG, in comparison to the procedure doe 36 hours after hCG administration.

In our study, the difficulty in the IUI procedure decreases CPR; however, other studies show no differences in CPR for an easy or difficult IUI (Khan *et al*., 2013). From all factors associated with IUI evaluated in the present study, we concluded that the following are those that keep a higher association with the success of the procedure: to have ≥2 follicles (OR 2.873), to achieve an IUI without difficulty (2.341), to have ≤3 years of infertility (OR 2.240), cervical factor as indication (OR 2.194), use of gonadotropins (OR 1846) and age ˂38 years (OR 1819).

## CONCLUSION

IUI is part of the initial management of infertility and demonstrates acceptable pregnancy rates when it is accompanied by proper selection and couples' preparation. This study shows those factors with more chance to be associated with pregnancy in IUI: to have ≥2 follicles, to achieve an IUI without difficulty, to have ≤3 years of infertility, cervical factor as indication, use of gonadotropins and age <38 years.

## References

[r1] Alborzi S, Motazedian S, Parsanezhad M, Jannati S (2003). Comparison of the effectiveness of single intrauterine insemination (IUI) versus double IUI per cycle in infertile patients. Fertil Steril.

[r2] Ashrafi M, Rashidi M, Ghasemi A, Arabipoor A, Daghighi S, Pourasghari P, Zolfaghari Z (2013). The role of infertility etiology in success rate of intrauterine insemination cycles: an evaluation of predictive factors for pregnancy rate. Int J Fertil Steril.

[r3] Banker M, Patel A, Deshmukh A, Shah S (2018). Comparison of Effectiveness of Different Protocols Used for Controlled Ovarian Hyperstimulation in Intrauterine Insemination Cycle. J Obstet Gynaecol India.

[r4] Bensdorp AJ, Tjon-Kon-Fat RI, Bossuyt PM, Koks CA, Oosterhuis GJ, Hoek A, Hompes PG, Broekmans FJ, Verhoeve HR, de Bruin JP, van Golde R, Repping S, Cohlen BJ, Lambers MD, van Bommel PF, Slappendel E, Perquin D, Smeenk JM, Pelinck MJ, Gianotten J, Hoozemans DA, Maas JW, Eijkemans MJ, van der Veen F, Mol BW, van Wely M (2015). Prevention of multiple pregnancies in couples with unexplained or mild male subfertility: randomised controlled trial of in vitro fertilisation with single embryo transfer or in vitro fertilisation in modified natural cycle compared with intrauterine insemination with controlled ovarian hyperstimulation. BMJ.

[r5] Boomsma CM, Heineman MJ, Cohlen BJ, Farquhar C (2007). Semen preparation techniques for intrauterine insemination. Cochrane Database Syst Rev.

[r6] Cabry-Goubet R, Scheffler F, Belhadri-Mansouri N, Belloc S, Lourdel E, Devaux A, Chahine H, De Mouzon J, Copin H, Benkhalifa M (2017). Effect of Gonadotropin Types and Indications on Homologous Intrauterine Insemination Success: A Study from 1251 Cycles and a Review of the Literature. Biomed Res Int.

[r7] Cantineau AE, Heineman MJ, Cohlen BJ (2003). Single versus double intrauterine insemination (IUI) in stimulated cycles for subfertile couples. Cochrane Database Syst Rev.

[r8] Cantineau AE, Janssen MJ, Cohlen BJ, Allersma T (2014). Synchronised approach for intrauterine insemination in subfertile couples. Cochrane Data Syst Rev.

[r9] Dyer S, Chambers GM, de Mouzon J, Nygren KG, Zegers-Hochschild F, Mansour R, Ishihara O, Banker M, Adamson GD (2016). International Committee for Monitoring Assisted Reproductive Technologies world report: Assisted Reproductive Technology 2008, 2009 and 2010. Hum Reprod.

[r10] ESHRE Capri Workshop Group (2009). Intrauterine insemination. Hum Reprod Update.

[r11] Farquhar C, Liu E, Armstrong S, Arroll N, Lensen S, Brown J (2017). A randomized controlled trial of intrauterine insemination with clomiphene citrate stimulation compared with expectant management for women with unexplained infertility (The TUI study). Hum Reprod.

[r12] Huang FJ, Chang SY, Lu YJ, Kung FT, Tsai MY, Wu JF (2000). Two different timings of intrauterine insemination for non-male infertility. J Assist Reprod Genet.

[r13] Irani M, Chow S, Keating D, Elder S, Rosenwaks Z, Palermo G (2018). Optimizing the first-line fertility treatment. Gynecol Endocrinol.

[r14] Khan SN, Storer BL, Peck JD, Goddard LD, Hansen KR, Craig LB (2013). Does a difficult intrauterine insemination lower pregnancy rates and live birth rates?. Fertil Steril.

[r15] Kuriya A, Agbo C, Dahan M (2018). Do pregnancy rates differ with intra-uterine insemination when different combinations of semen analysis parameters are abnormal?. J Turk Ger Gynecol Assoc.

[r16] Lee J, Hwang S, Lee J, Yoo J, Jang D, Hwang K, Kim M (2018). Effect of insemination timing on pregnancy outcome in association with female age, sperm motility, sperm morphology and sperm concentration in intrauterine insemination. J Obstet Gynaecol Res.

[r17] NICE - National Institute for Health and Care Excellence (2013). Fertility problems: assessment and treatment. https://www.nice.org.uk/guidance/cg156?unlid=86583397720167208641.

[r18] Nuojua-Huttunen S, Tomas C, Bloigu R, Tuomivaara L, Martikainen H (1999). Intrauterine insemination treatment in subfertility: an analysis of factors affecting outcome. Hum Reprod.

[r19] Punjabi U, De Neubourg D, Van Mulders H, Cassauwers W, Peeters K (2018). Validating semen processing for an intrauterine program should take into consideration the inputs, actions and the outputs of the process. Andrologia.

[r20] Ragni G, Maggioni P, Guermandi E, Testa A, Baroni E, Colombo M, Crosignani PG (1999). Efficacy of double intrauterine insemination in controlled ovarían hyperstimulation cycles. Fertil Steril.

[r21] Rahman SM, Karmakar D, Malhotra N, Kumar S (2011). Timing of intrauterine insemination: an attempt to unravel the enigma. Arch Gynecol Obstet.

[r22] Sicchieri F, Silva AB, Silva ACJSRE, Navarro PAAS, Ferriani RA, Reis RMD (2018). Prognostic factors in intrauterine insemination cycles. JBRA Assist Reprod.

[r23] Tonguc E, Var T, Onalan G, Altinbas S, Tokmak A, Karakas N, Gulerman C (2010). Comparison of the effectiveness of single versus double intrauterine insemination with three different timing regimens. Fertil Steril.

[r24] van Rumste MM, Custers IM, van der Veen F, van Wely M, Evers JL, Mol BW (2008). The influence of the number of follicles on pregnancy rates in intrauterine insemination with ovarian stimulation: a meta-analysis. Hum Reprod Update.

[r25] Wang YC, Chang YC, Chen IC, Cnien HH, Wu GJ (2006). Comparison of timing of IUI in ovarian stimulated cycles. Arch Androl.

[r26] Zahiri Sorouri Z, Rashid Shomali R, Pourmarzi D (2016). Single versus Double Intrauterine Insemination in Controlled Ovarian Hyperstimulation Cycles: A Randomized Trial. Arch Iran Med.

[r27] Zegers-Hochschild F, Schwarze JE, Crosby JA, Musri C, Urbina MT, Latin American Network of Assisted Reproduction (REDLARA) (2016). Assisted reproductive techniques in Latin America: the Latin American Registry, 2013. JBRA Assist Reprod.

